# Improving the timeliness of birth registration in Fiji through a financial incentive

**DOI:** 10.1016/j.gloepi.2024.100162

**Published:** 2024-09-10

**Authors:** Christine Linhart, Neel Singh, Meli Nadakuca, Varanisese Saumaka, Carlie Congdon, Sharita Serrao, Richard Taylor, Stephen Morrell

**Affiliations:** a School of Population Health, University of New South Wales, Samuels Building, Botany St, UNSW, Sydney, NSW 2052, Australia; b Civil Registry, Ministry of Justice, Suvavou House, Victoria Parade, Suva, Fiji; c Fiji Bureau of Statistics, Modyl Plaza, Lot 1 Karsanji Street Vatuwaqa, Suva, Fiji; d Fiji Ministry of Health and Medical Services, 88 Amy Street, Toorak, Suva, Fiji; e Vital Strategies, 100 Broadway, 4th Floor, New York, USA; f United Nations Economic and Social Commission for the Asia Pacific, Rajdamnern Nok Avenue, Bangkok, Thailand

**Keywords:** Fiji, Birth registration, Timeliness, Financial incentive, Ethnicity, Adolescent, Marital status

## Abstract

**Background:**

Fiji is a Pacific Island nation with the predominant ethnic groups indigenous Fijians (iTaukei) (62 %) and Fijians of Indian descent (31 %). This study reports on the effect of a Parental Assistance Payment Program (PAPP) tied to on-time birth registration, available in Fiji from August 2018 to July 2020.

**Methods:**

Unit record birth registration data (*n* = 117,829) for children born during 2016–22 were used to calculate mean birth-to-registration intervals and the likelihood of on-time birth registration (within 365 days) before the PAPP (January 2016–July 2018) compared to during the PAPP (August 2018–July 2020), by population disaggregations (sex, ethnicity, age, marital status).

**Results:**

During the PAPP, mean birth-to-registration intervals declined sharply by 81 %, from 665 days (95 %CI: 658–671) to 124 days (121–127). The largest declines were among i-Taukei children (803 to 139 days, 83 %) compared to non-iTaukei (283 to 76 days, 73 %); mothers aged 10–19 years (880 to 134 days, 85 %) compared to ≥20 years (653 to 123 days, 81 %); and single mothers (983 to 145 days, 85 %) compared to married mothers (570 to 115 days, 80 %). On-time birth registration increased from 57 % to 93 %, and the adjusted hazard ratio showed children born during the PAPP were 2.3 times more likely (95 %CI: 2.2–2.4) to have their birth registered on-time compared to children born before the PAPP. When the PAPP was discontinued in August 2020, the birth-to-registration interval increased sharply in all population groups.

**Conclusions:**

During the two-year period the PAPP was available, it was highly effective at improving the timeliness of birth registration, particularly among iTaukei children, young mothers, and single mothers. After the PAPP was discontinued, the timeliness of birth registration deteriorated sharply. Longer post-PAPP follow-up time (≠5 years) is required to determine whether the timeliness of birth registration has deteriorated to levels similar to those during the pre-PAPP period.

## Background

Birth registration gives individuals a legal identity and rights to access benefits and legal protections afforded by the state. These include access to education and medical care, the ability to open a bank account and obtain a passport, and the right to vote. Complete and timely birth registration provides data for a continuous picture of fertility trends and child mortality rates in a country, which are fundamental for monitoring and national planning across multiple sectors. Health planners and policy makers use these data to facilitate the allocation of resources for antenatal and postnatal services. The education sector uses birth registration data to estimate the resources required for schools, and to monitor rates of school enrolment and completion. Government ministries responsible for infrastructure, including transport, housing, water and land resources, depend on accurate population data to plan for current and future populations. At a regional and global level, the importance of birth registration is well recognised in the 2030 Agenda for Sustainable Development, with the aim of providing legal identity for all, including birth registration, a target in and of itself (16.9) [1].

Disparities often exist in the completeness and timeliness of birth registration among different population groups. The magnitude of the disparity, however, is often unknown due to a lack of birth registration data disaggregated by, for example, the mothers age, ethnicity, education, income level, or marital status. The Ministerial Declaration to “Get Every One in The Picture” in Asia and the Pacific [2], and the Regional Action Framework (RAF) on Civil Registration and Vital Statistics (CRVS) in Asia and the Pacific [3], recognise the need to identify and address disparities in the completeness and timeliness of birth registration across different population groups.

The Republic of Fiji Islands forms part of Melanesia in the South Pacific. According to the most recent Fiji census of population and housing in 2017, the population was estimated to be 884,887, with the predominant ethnic groups indigenous Fijians (iTaukei) (63 %) and Fijians of Indian descent (33 %). The most recent patterns in fertility in Fiji, from empirical data on births, show that during 2016–19 the total fertility rate varied between 2.6 and 2.8 births per woman [4]. Age-specific fertility was highest among women aged 25–29 years (157–171 births per 1000 women), followed by women 20–24 years (148–156/10^3^), then 30–34 years (113–123/10^3^). Fertility declined sharply at 35–39 years (60–67/10^3^) and further by 40–44 years (20–21/10^3^). The adolescent fertility rate (15–19 years) showed a consistent gradual increase during 2016–19, from 26 births per 1000 in 2016 to 34 births per 1000 in 2019 [4].

The Ministry of Justice is responsible for overseeing birth registration in Fiji, with the National Births, Deaths and Marriages (BDM) Office located in Suva, and a further 20 BDM offices across Fiji. Birth registration is governed by the Fiji Births, Deaths and Marriages Registration Act of 1975. The Act states that births should be registered within two months of the child's birth, but birth registration is considered ‘on-time’ if completed within one year of the child's birth [5].

From August 1, 2018, to July 31, 2020, the Fiji Government introduced a financial incentive tied to on-time birth registration called the Parental Assistance Payment Program (PAPP), which aimed to provide some support to low-income families with the costs of raising a child. Parents could access the program if: their child was born during the two-year period the PAPP was available; birth registration was completed within 12 months of the birth; and the combined annual parental income was ≤30,000 Fijian dollars (FJ$) (approximately ≤USD $13,000). A PAPP application form needed to be completed at a BDM office at the time of birth registration, which included a combined parental income declaration. The birth certificate and completed PAPP application would then be taken to a bank of the parent's choice, and the Ministry of Finance deposited FJ$500 into the parent's bank account immediately, with an additional FJ$500 deposited into a bank account opened in the child's name to be made available to parents when the child enrolled in primary school. Audit teams from the Ministry of Finance were responsible for spot checks to verify that PAPP claims and declarations were accurate. Based on the number of PAPP applications completed by BDM offices (*n* = 29,695), parents of 78 % of children born during the two-year PAPP period applied for the PAPP.

To date, no national evaluation of the effectiveness of the PAPP on the timeliness of birth registration in Fiji by population disaggregations has been undertaken. The objectives of the present study were to determine the effect of the PAPP on the timeliness of birth registration in Fiji by sex and ethnicity of the child (iTaukei and non-iTaukei), and by maternal age and marital status.

## Methods

### Data sources

#### Birth registrations

Birth registration data for children born during 2016–22 were extracted from the Fiji Ministry of Justice database on 5th January 2024 and contained 117,842 individual birth registration unit records. The variables used for analysis in relation to the child were sex (male or female), ethnicity (iTaukei or non-iTaukei), date of birth and date of registration of birth, and the mother's date of birth and marital status at the time of registration of the child (single, married, divorced/widowed). These variables had a valid entry for ≥99 % of all unit records. For children born while the PAPP was available, the unit record dataset did not contain a variable that enabled identification of the 78 % of unit records which had applied for the PAPP, and the 22 % which had not.

Ethnicity of the child, as reported by the mother, was recorded as either iTaukei or non-iTaukei, and had a valid entry for all unit records during 2016–22. Completeness of the variable for mother's ethnicity varied greatly by individual year and was deemed unreliable for analysis. For 2019–22, where completeness of the mother's ethnicity variable exceeded 98 %, among the children whose ethnicity was recorded as iTaukei, 95 % also had their mother's ethnicity recorded as iTaukei, 4.2 % were recorded as non-iTaukei and 1.3 % had no ethnicity recorded. Ethnicity of the child is thus a reasonable surrogate for ethnicity of the mother. Ethnicity of the father is not recorded in the birth registration dataset.

#### Health service data

The Ministry of Health and Medical Services (MHMS) has two databases that record the number of births in Fiji: (1) the national unit record electronic database (PATISPlus), which records information contained in notification of birth forms; and (2) the health service utilisation reporting system (known as the Consolidated Monthly Reporting Information System (CMRIS)), which requires all health facilities to report the monthly aggregate birth numbers (both sexes combined) recorded in the hard-copy birth ledger of each health facility. Zone nurses report any community births which occur outside health facilities in their monthly reports, but these are noted to be rare events [6]. The aggregate number of births reported by individual health facilities through the CMRIS varied minimally each year during 2016–22, indicating that it is a well-functioning system for capturing the aggregate annual number of births in Fiji [4]. By comparison, a recent estimation of the completeness of the national notification of birth unit record database (PATISPlus) found it to vary widely during 2016–2022 from 28 % to 80 % when compared to the health service utilisation reporting system (CMRIS) [4]. For this reason, the CMRIS birth dataset was selected for use as the denominator for assessing the overall completeness of birth registration in the present study.

### Analysis

The main outcomes of interest for comparing the periods before PAPP and during PAPP were: (1) the mean birth-to-registration interval (days between date of birth and date of birth registration); (2) the percentage of births registered on-time (≤365 days); and (3) the hazard ratio of on-time birth registration using multiple Cox proportional hazards regression analyses (univariable unadjusted, and multivariable adjusted with and without interaction terms). These analyses were performed according to sex and ethnicity of the child, and maternal age and marital status. Since preliminary analysis showed the mean birth-to-registration interval was not significantly different by 10-year age group among mothers aged 20–49 years, these age groups were combined. Interaction terms in the proportional hazards regression models of the PAPP by the subgroups of interest were used to quantify differential effects of the PAPP on these groups, with 95 % confidence intervals and levels of statistical significance. Records were not available of children whose birth had not been registered during the study period, including those who may have died before registration. As a consequence, the analysis is confined to factors associated with timely birth registration.

The accuracy of mean birth-to-registration intervals and the percentage of births registered on-time calculated in this study are dependent on the completeness of birth registration data. When completeness is high (≥95 %) they provide an accurate indication of the timeliness of birth registration, but when completeness is lower, they overestimate birth registration timeliness because a large number of late birth registrations are missing from the dataset. Annual birth registration completeness for 2016–22 was estimated by dividing the number of births by year of birth as recorded in the Ministry of Justice birth registration dataset (numerator) by the corresponding number of births in the MHMS CMRIS aggregate birth dataset (denominator). The estimated annual completeness of birth registration data for 2016–19 was ≥95 %, declining to 80.9 % for 2020, 61.7 % for 2021 and 52.0 % for 2022. Lower birth registration rates post 2020 are partly artefactual due to the shorter available follow-up time.

Accordingly, for the period following discontinuation of the PAPP (August 2020 onwards), mean birth-to-registration intervals and the percentage of births registered on-time were not calculated because of this decline in birth registration completeness. However, hazard ratios of on-time birth registration (univariate unadjusted, and multivariable adjusted with and without interaction terms) for August 2020 to December 2022 were calculated, and mean birth-to-registration intervals for August to December 2020 are displayed (as broken lines) to indicate the initial effect of the discontinuation of the PAPP in August 2020 on the mean-birth-to registration interval.

SAS version 9.4 (SAS Institute Inc., Cary, NC) was used for all analyses.

## Results

**Fig. 1:**
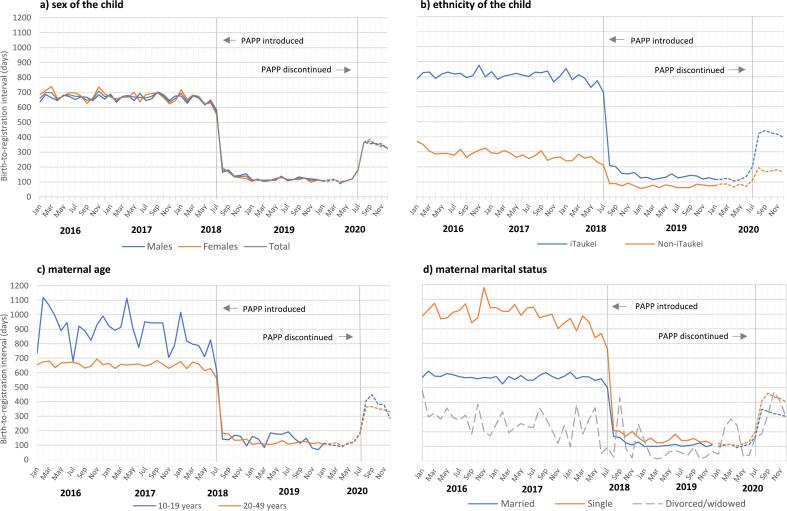
Mean birth-to-registration interval by month of birth, by sex and ethnicity of the child and by maternal age and marital status, 2016–20. The mean monthly intervals for 2020 may be significantly underestimated because of the lower completeness of the birth registration data in 2020 (81 %) compared to 2016–19 (≥95 %). Given the clear effect of the PAPP for 2018–19, it would be expected that this lower completeness would apply more to children born in the latter (August–December post-PAPP) part of 2020, and the data are displayed as broken lines for this period only to show the initial effect that the discontinuation of the PAPP had on the timeliness of birth registration. The estimates for the 2020 post-PAPP period can be expected to increase somewhat over time, as unregistered children become registered. The divorced/widowed category contains very low numbers and is therefore prone to stochastic variation.

The PAPP sharply reduced the overall mean birth-to-registration interval by 81 %, from 665 to 124 days, and increased the proportion of births registered on-time from 57 % to 93 % (Table 1, Fig. 1a). The unadjusted hazard ratio for on-time registration showed that children born during the PAPP were 4.1 times more likely to have their birth registered on-time compared to children born prior to the PAPP. After adjusting for the sex and ethnicity of the child, the maternal age and marital status, and the differential effects of the PAPP on these subgroups (as shown by the regression model interaction terms), the hazard ratio was 2.3 (Table 2).

**Table 1:** Timeliness of birth registration among children born before, during and after the introduction of the PAPP.

Factors/variables	n (%)	Mean days 95 %CI	On-time (%)	Unadjusted HR (95 % CI)	Adjusted HR ^ (95 % CI)
**Children born before the PAPP - January 2016 to July 2018**					
**All**	50,152	665 (658–671)	57		
**Child sex**					
Male	26,098 (52)	660 (651–669)	57	Ref	Ref
Female	24,054 (48)	670 (661–680)	57	0.99 (0.97–1.01)	0.98 (0.96–1.00)
**Child's ethnicity**					
Non-iTaukei	13,355 (27)	283 (274–291)	83	Ref	Ref
iTaukei	36,797 (73)	803 (796–811)	48	0.35 (0.35–0.36)**	0.38 (0.37–0.39)**
**Maternal age group**					
≥20 years	47,461 (95)	653 (646–659)	58	Ref	Ref
10–19 years	2691 (5)	880 (850–910)	44	0.66 (0.62–0.70)**	0.82 (0.78–0.87)**
**Maternal marital status**					
Married	37,037 (74)	570 (563–577)	63	Ref	Ref
Single	12,190 (24)	983 (969–997)	39	0.48 (0.47–0.50)**	0.55 (0.53–0.56)**
Divorced/widowed	919 (2)	276 (252–301)	80	1.40 (1.31–1.50)**	1.13 (1.05–1.21)*
**Children born during the PAPP - August 2018 to July 2020**					
**All**	38,098	124 (121–127)	93		
**Child sex**					
Male	19,675 (52)	124 (120–129)	93	Ref	Ref
Female	18,423 (48)	123 (119–18)	93	1.01 (0.99–1.03)	1.01 (0.99–1.03)
**Child's ethnicity**					
Non-iTaukei	8994 (24)	76 (72–80)	96	Ref	Ref
iTaukei	29,104 (76)	139 (135–142)	92	0.83 (0.81–0.84)**	0.84 (0.82–0.86)**
**Maternal age group**					
≥20 years	35,661 (94)	123 (120–126)	93	Ref	Ref
10–19 years	2437 (6)	134 (122–146)	93	0.93 (0.89–0.96)**	1.00 (0.96–1.04)
**Maternal marital status**					
Married	26,507 (70)	115 (111–118)	93	Ref	Ref
Single	11,443 (30)	145 (139–151)	92	0.84 (0.82–0.86)**	0.86 (0.84–0.88)**
Divorced/widowed	144 (0)	101 (63–139)	94	1.04 (0.87–1.24)	1.04 (0.88–1.24)
**Children born after the PAPP - August 2020 to December 2022**					
**All**	29,579	–	–		
**Child sex**					
Male	15,344	–	–	Ref	Ref
Female	14,235	–	–	0.97 (0.95–1.00)*	0.97 (0.95–1.00)*
**Child's ethnicity**					
Non-iTaukei	7782	–	–	Ref	Ref
iTaukei	21,797	–	–	0.52 (0.51–0.54)**	0.53 (0.51–0.55)**
**Maternal age group**					
≥20 years	27,973	–	–	Ref	Ref
10–19 years	1606	–	–	0.90 (0.85–0.95)*	0.97 (0.91–1.03)
**Maternal marital status**					
Married	21,518	–	–	Ref	Ref
Single	7995	–	–	0.76 (0.74–0.78)**	0.80 (0.77–0.82)**
Divorced/widowed	64	–	–	0.91 (0.70–1.20)	0.78 (0.59–1.04)

PAPP = Parental Assistance Payment Program; n = number of birth registrations of children born and registered prior to the PAPP and during the PAPP; HR = hazard ratio; Mean days (95 %CI) = mean number of days between the recorded date of birth and date of birth registration, with 95 % confidence intervals; *p < 0.05; **p < 0.0001; On-time (%) = the proportion of children registered within 365 days of birth; Ref = reference group (OR 1.00); ^ adjusted for sex and ethnicity of child, and maternal age and marital status; marital status blank for 6 records before the PAPP, 4 records during the PAPP and 2 records after the PAPP.

**Table 2:** Overall effect of the PAPP during the PAPP (August 2018 to July 2020) compared to before the PAPP (January 2016 to July 2018).

Variables and interactions	HR (95 % CI)
**Univariable unadjusted analysis**	
Born during PAPP (cf. before)	4.10 (4.03–4.17)**
**Multivariable adjusted**^**^**^**analysis with interaction terms**	
Birth period = born during PAPP (cf. born before PAPP)	2.30 (2.22–2.37)**
Child sex = female (cf. male)	0.98 (0.96–1.00)
Born during PAPP*female	1.03 (0.99–1.06)
Child ethnicity = iTaukei (cf. non-iTaukei)	0.41 (0.40–0.42)**
Born during PAPP*iTaukei	1.93 (1.87–2.00)**
Maternal age group = 10–19 years (cf. ≥20 years)	0.83 (0.79–0.88)**
Born during PAPP*10–19 years	1.20 (1.12–1.30)**
Maternal marital status = single (cf. married)	0.57 (0.55–0.59)**
Maternal marital status = divorced/widowed (cf. married)	1.13 (1.06–1.20)*
Born during PAPP*single	1.49 (1.43–1.56)**
Born during PAPP*divorced/widowed	0.92 (0.74–1.14)

PAPP = Parental Assistance Payment Program; HR = hazard ratio; 95 %CI = 95 % statistical confidence intervals; *p < 0.05; **p < 0.0001; ^adjusted for sex and ethnicity of child, and maternal age and marital status.

### Sex of the child

Among male children, the PAPP sharply reduced the mean birth-to-registration interval from 660 to 124 days (81 % decline), and among female children from 670 to 123 days (82 % decline); while on-time birth registration increased from 57 % to 93 % in both sexes (Table 1, Fig. 1a). Adjusted hazard ratios showed that there was no notable sex-specific difference in on-time birth registration between female and male children prior to the PAPP (HR = 0.98) or during the PAPP (HR = 1.01) (Table 1).

### Ethnicity of the child

Among i-Taukei children, the PAPP sharply reduced the mean birth-to-registration interval from 803 to 139 days (83 % decline), and among non-iTaukei children from 283 to 76 days (73 % decline); while on-time birth registration increased from 48 % to 92 % in i-Taukei children, and from 83 % to 96 % in non-iTaukei children (Table 1, Fig. 1b). Adjusted hazard ratios showed that prior to the PAPP, on-time birth registration was 62 % less likely among iTaukei compared to non-iTaukei children (HR = 0.38), with the disparity reducing to 17 % during the PAPP (HR = 0.84) (Table 1).

### Maternal age

Among mothers aged 10–19 years, the PAPP sharply reduced the mean birth-to-registration interval from 880 to 134 days (85 % decline), and among mothers ≥20 years from 653 to 123 days (81 % decline); while on-time birth registration increased from 44 % to 93 % in mothers aged 10–19 years, and from 58 % to 93 % in mothers ≥20 years (Table 1, Fig. 1c). Adjusted hazard ratios showed that prior to the PAPP, on-time birth registration was 18 % less likely among mothers aged 10–19 years compared to mothers ≥20 years (HR = 0.82), whilst during the PAPP no age-specific disparity in on-time registration was evident (HR = 1.00) (Table 1).

### Maternal marital status

The PAPP sharply reduced the mean birth-to-registration interval among single mothers from 983 to 145 days (85 % decline); married mothers from 570 to 115 days (80 % decline); and divorced/widowed mothers from 276 to 101 days (63 % decline). While on-time birth registration increased from 39 % to 92 % in single mothers; 63 % to 93 % in married mothers; and 80 % to 94 % in divorced/widowed mothers (Table 1, Fig. 1d). Prior to the PAPP, adjusted hazard ratios showed that on-time birth registration was 45 % less likely among single mothers compared to married mothers (HR = 0.55), with the disparity decreasing to 14 % during the PAPP (HR = 0.86) (Table 1).

### Post-PAPP period

The unadjusted hazard ratio for on-time registration showed that children born during the PAPP were 3.9 times more likely (95 %CI: 3.8–4.0) to have their birth registered on-time compared to children born after the PAPP was discontinued (Table 3). After adjusting for the sex and ethnicity of the child, the maternal age and marital status, and the differential effects of the PAPP on these subgroups (as shown by the regression model interaction terms), the hazard ratio was 2.1 (95 %CI: 2.0–2.1). After the PAPP was discontinued, adjusted hazard ratios showed that a large disparity in on-time birth registration was again present among iTaukei children compared to non-iTaukei children and single mothers compared to married mothers (Table 1). A small disparity emerged between female children compared to male children, whilst among younger mothers compared to older mothers no disparity in on-time registration was evident.

**Table 3:** Overall effect of the PAPP during the PAPP (August 2018 to July 2020) compared to after the PAPP (August 2020 to December 2022).

Variables and interactions	HR (95 % CI)
**Univariable unadjusted analysis**	
Born during PAPP (cf. after)	3.90 (3.84–3.96)**
**Multivariable adjusted**^**^**^**analysis with interaction terms**	
Birth period = born during PAPP (cf. born after PAPP)	2.06 (1.99–2.13)**
Child sex = female (cf. male)	0.97 (0.95–1.00)*
Born during PAPP*female	1.03 (1.00–1.07)
Child ethnicity = iTaukei (cf. non-iTaukei)	0.59 (0.58–0.61)**
Born during PAPP*iTaukei	1.33 (1.28–1.38)**
Maternal age group = 10–19 years (cf. ≥20 years)	0.97 (0.92–1.02)
Born during PAPP*10–19 years	1.03 (0.96–1.11)
Maternal marital status = single (cf. married)	0.83 (0.81–0.85)**
Maternal marital status = divorced/widowed (cf. married)	0.83 (0.65–1.05)
Born during PAPP*single	1.03 (0.99–1.07)
Born during PAPP*divorced/widowed	1.25 (0.91–1.72)

PAPP = Parental Assistance Payment Program; HR = hazard ratio; 95 %CI = 95 % statistical confidence intervals; *p < 0.05; **p < 0.0001; ^adjusted for sex and ethnicity of child, and maternal age and marital status.

## Discussion

The introduction of a financial incentive scheme tied to on-time birth registration (the PAPP) from August 2018 until July 2020 had immediate and substantial effects on improving the timeliness of birth registration in Fiji throughout the two-year period during which the payment was available. Among children born from January 2016 to July 2018, prior to the introduction of the PAPP, the average time for a child's birth to be registered was almost two years (665 days) and only 57 % of births were registered on-time (≤365 days). The most significant delay in birth registration during the pre-PAPP period was among iTaukei children compared to non-iTaukei children, followed by single mothers compared to married mothers, and then younger mothers (10–19 years) compared to older mothers (≥20 years). Female children were found to have greater delays in birth registration than male children, however, the difference was very small. Following the introduction of the PAPP, the average time for a child's birth to be registered declined sharply to approximately four months (124 days), and 93 % of births were registered on time. The greatest improvements in the timeliness of birth registration were among iTaukei children, mothers aged 10–19 years, and single mothers. During the PAPP, the disparity in on-time birth registration between iTaukei and non-iTaukei children was reduced from 62 % to 17 %, in single mothers compared to married mothers from 45 % to 14 %, and between young mothers compared to older mothers from 18 % to 0 %.

When the PAPP was discontinued in August 2020, the birth-to-registration interval increased sharply to approximately half of the pre-PAPP level. While this may indicate a long-term positive effect of the PAPP on social and cultural norms concerning on-time birth registration, it is also partly explained by artefact due to lower completeness of birth registration data during the shorter post-PAPP follow-up period. For 2020, where birth registration completeness was estimated at 82 %, the mean birth-to-registration intervals can be expected to increase over time, as unregistered children become registered. Given the clear effect of the PAPP for 2018–19, it would be expected that this would apply more to children born in the latter (August–December post-PAPP) part of 2020. Therefore, a longer follow-up time is required for birth registration completeness to reach ≥95 % for 2020 onwards before it can be determined whether the mean birth-to-registration intervals during the post-PAPP period return to similar levels as those before the PAPP's introduction, and the extent to which a possible residual effect of the PAPP on timely birth registration exists. For this reason, analysis of mean birth-to-registration intervals and the percentage of births registered on-time birth for the post-PAPP period were not undertaken in this study due to a lack of sufficient follow-up time (≠5 years). Nonetheless, a residual effect of the PAPP is evidenced by lower ethnic, age and marital status disparities in likelihood of birth registration in the post-PAPP period compared to the pre-PAPP period, as indicated by the higher estimated hazard ratios for the post-PAPP compared to the pre-PAPP period for iTaukei mothers, mothers <20 years and for single mothers (Table 2, Table 3).

We estimated the national completeness of birth registration during 2016–17, prior to the introduction of the PAPP, as ≥95 %. While high levels of completeness can be achieved in the absence of financial incentives, the mean birth-to-registration interval during 2016–17 was almost two years, whereas during the PAPP (August 2018 to July 2020), the mean birth-to-registration interval was four months. In the absence of the PAPP, the main requirements for birth registration and a birth certificate in Fiji are primarily for school enrolment or for obtaining a child's passport. These are not linked to timely birth registration, with school enrolment not occurring before the child is at least four years of age, and with many children never obtaining a passport. In the absence of incentives tied to timely birth registration, such as the PAPP, it could be expected to take five or more years for the national completeness of birth registration in Fiji to reach ≥95 %. For instance, among children born in 2020, birth registration completeness may not reach ≥95 % until 2025, once those children have reached primary school age and require a birth certificate for enrolment.

An increase in birth registration completeness when children are around five years of age has been documented in many countries where birth registration is required for school enrolment, and is often the first incentive for parents to undertake the registration process. This has been observed in Nepal [7], India [8], Uganda [9], and Nigeria [10]. Delayed birth registration, however, leaves a child without a legal identity and has been shown to increase a child's risk of statelessness, trafficking, and child labour [8,11,12] and exclusion from essential services including health care [13,14]. Delayed birth registration also, importantly, hinders the availability of complete and timely vital statistics on fertility and child mortality that are essential for accurate monitoring and national planning across multiple government sectors, or for reporting progress against global development agenda frameworks such as the Sustainable Development Goals.

The largest disparity in the timeliness of birth registration before, during and after the PAPP was between iTaukei children and non-iTaukei children. The reasons for this disparity could be diverse and need to be better understood. The 2021 Fiji Multiple Indicator Cluster Survey (MICS) found that wealth quintile was positively associated with increased birth registration, with the proportion of children under age 5 reported to have their birth registered estimated at 78 % among the poorest wealth quintile, rising to 95 % among the richest quintile [15]. The intersection between wealth quintile and ethnicity, however, is not well known in Fiji, with no ethnicity-specific disaggregations in the 2021 MICS survey [15], nor in the 2019 Fiji Household Income and Expenditure Survey [16]. It is possible that mothers of iTaukei children are more likely to be in the lower wealth quintile, and thus poverty may have a greater impact on birth registration due to direct and indirect costs, rather than cultural aspects of ethnicity.

Furthermore, geography may play a part since the iTaukei population live in more rural and remote areas than the non-iTaukei population [17], with greater transport barriers in accessing civil registration offices. Standardisation of geographic birth registration data (currently being undertaken by the civil registry) and disaggregation of household income and expenditure data by ethnicity, will facilitate a better understanding of the current ethnic-specific disparities in the timeliness of birth registration in Fiji. However, the large reduction in the ethnic disparity in birth registration associated with the PAPP suggests lower mean income levels in iTaukei compared to non-iTaukei may be a factor in the pre- and post-PAPP ethnic disparity in on-time birth registration.

Direct and indirect costs associated with birth registration are well documented barriers for parents and caregivers completing the birth registration process [18]. Many studies have identified that loss of wages and transportation costs are the main barriers to birth registration, particularly among poorer households [8]. Financial incentives have been shown to be effective in increasing birth registration through a variety of mechanisms in Asia, Africa and Latin America [19]. The financial incentives have either directly aimed to improve birth registration timeliness and completeness, or have included birth registration as an administrative requirement for access to other financial incentive programs or schemes. In India, the implementation of the Majoni scheme in 2009 provided financial incentives for the registration of female children, and within one-year female birth registration increased from 24 % to 39 % in the target population [20]. In Zimbabwe, the implementation of a cash transfer program in 2010, which mandated birth registration as a condition of enrolment in the scheme, documented an increase in birth registration from 8 % to 25 % within one year among children aged 0–4 years in the target population [21]. South Africa's Child Support Grant is a nationwide cash transfer to households under the government-determined income threshold, and has been cited as a significant contributor to increasing South Africa's birth registration from 21 % in 1992 to 84 % in 2012 [22].

Greater delays in birth registration among younger mothers compared to older mothers were identified in this study prior to the introduction of the PAPP. Whereas during the PAPP, no age-specific disparity in on-time registration was evident, again suggesting that income may play a role as a barrier to birth registration. Previous studies in the Asia-Pacific region have identified greater delays in birth registration among younger mothers, however, possible factors influencing these delays have not been proposed [23,24]. By contrast, a recent review of birth registration in five countries in East and Southern Africa, based on Demographic and Health Survey (DHS) data, reported limited evidence of early childbearing negatively affecting the likelihood of birth registration [25]. Better understanding is needed of the barriers and facilitators to on-time birth registration among young mothers in Fiji.

### Limitations

The dataset used in this study did not enable individual identification of which birth registrations also accompanied an application for the PAPP (78 % overall), and which did not (22 %), during the two-year period the PAPP was available. Exceeding the combined parental income threshold likely explains a portion of the latter category. Although the intersection between wealth quintile and ethnicity is not well understood in Fiji, married mothers who had to declare both parents' income, compared with single mothers, were likely to constitute a larger portion of the group that exceeded the PAPP's income threshold. This may have resulted in a lower overall effect of the PAPP among married mothers than otherwise if this group had similar access to the PAPP.

The maternal marital status used in this study was recorded at the time of birth registration. When birth registration is delayed, particularly by several years, the maternal marital status may be different to what it was at the time of giving birth. In this study, the number of married women was 10 % lower during the PAPP compared to before the PAPP, which suggests that before the PAPP up to 10 % of women in the married category may have been single at the time of giving birth. The effect of this proportion of potentially misclassified marital status records would not be expected to substantially change the large difference in the timeliness of birth registration identified between single and married mothers.

## Conclusions

Fiji implemented an effective financial incentive scheme (the PAPP) from August 2018 to July 2020 that improved the timeliness of birth registration. Consequently, the proportion of children spending prolonged periods without a legal identity was reduced, and the availability and timeliness of complete vital statistics data were improved. The economic incentives provided through the PAPP had a particularly large positive impact on the iTaukei population, and on young mothers and single mothers who, prior to the PAPP, had considerably longer birth-to-registration intervals and significantly lower likelihood of registering within 12 months compared with older, married, and non-iTaukei mothers. The continuation of economic incentives should be considered to improve the completeness and timeliness of birth registration for all subsets of the population, with a particular focus on addressing the otherwise large disparity in birth registration between the iTaukei and the non-iTaukei population which exists in the absence of economic incentives. Even small economic incentives, or coupons to exchange for items required to care for a newborn, are likely to have a positive impact on birth registration and these options should be explored. Economic analyses of the PAPP and return-on-investment research may also provide further evidence of the potential benefits from the reinstatement of the PAPP or similar economic incentives tied to on-time birth registration.

## Ethics approval and consent to participate

This study was approved by the Fiji National Health Research and Ethics Review Committee (FNHRERC Number: 01/2024) and by the University of New South Wales Human Research Ethics Committee (iRECS5810). This study involved secondary use of existing data provided to the research team in a non-identifiable format which met the requirements for a waiver of consent as stipulated by the National Health and Medical Research Council of Australia. A waiver of consent for this study was approved by the UNSW HREAP Executive Committee.

## Consent for publication

Not applicable.

## Availability of data and materials

The birth registration dataset analysed in this study is not publicly available but may be requested upon submission of a data request to the Civil Registry in the Fiji Ministry of Justice. The nonconfidential information used in this publication was compiled in accordance with the 2018 Information Act of the Republic of Fiji, but which the Civil Registry has no authority to independently verify. The Civil Registry cannot and does not represent that the data was appropriate for this publication, or endorse or support any conclusions that may be drawn from the use of the data.

## Funding

Not applicable.

## CRediT authorship contribution statement

**Christine Linhart:** Writing – review & editing, Writing – original draft, Visualization, Validation, Supervision, Software, Resources, Project administration, Methodology, Investigation, Funding acquisition, Formal analysis, Data curation, Conceptualization. **Neel Singh:** Writing – review & editing, Methodology, Data curation, Conceptualization. **Meli Nadakuca:** Writing – review & editing, Methodology, Conceptualization. **Varanisese Saumaka:** Writing – review & editing, Methodology, Conceptualization. **Carlie Congdon:** Writing – review & editing, Methodology. **Sharita Serrao:** Writing – review & editing, Methodology. **Richard Taylor:** Writing – review & editing, Methodology. **Stephen Morrell:** Writing – review & editing, Writing – original draft, Validation, Methodology, Formal analysis, Conceptualization.

## Declaration of competing interest

The authors declare that they have no competing interests.
